# Efficacy and safety of tranexamic acid in patients undergoing thoracic surgery: a systematic review and PRISMA-compliant meta-analysis

**DOI:** 10.1186/s13019-024-02716-9

**Published:** 2024-04-09

**Authors:** Bin Gao, Yang Liu, Yun-tai Yao, the Evidence in Cardiovascular Anesthesia (EICA) Group

**Affiliations:** 1 Department of Anesthesiology, Huzhou Central Hospital, The Affiliated Central Hospital of Huzhou University, No. 1558, Sanhuan North Road, Huzhou, 313000 China; 2 Department of Anesthesiology, Fuwai Hospital, National Center for Cardiovascular Diseases, Peking Union Medical College and Chinese Academy of Medical Sciences, No. 167, Beilishi Road, Xicheng District, Beijing, 100037 China

**Keywords:** Tranexamic acid, Thoracic surgery, Bleeding, Transfusion

## Abstract

**Objectives:**

Perioperative bleeding poses a significant issue during thoracic surgery. Tranexamic acid (TXA) is one of the most commonly used antifibrinolytic agents for surgical patients. The purpose of the current study was designed to investigate the efficacy and safety of TXA in patients undergoing thoracic surgery.

**Methods:**

An extensive search of PubMed, Web of Science (WOS), Cochrane Library (trials), Embase, OVID, China National Knowledge Infrastructure (CNKI), Wanfang, and VIP electronic databases was performed to identify studies published between the inception of these databases and March 2023. The primary outcomes included perioperative blood loss and blood transfusions. Secondary outcomes of interest included the length of stay (LOS) in hospital and the incidence of thromboembolic events. Weighted mean differences (WMDs) or odds ratios (OR) with 95% confidence intervals (CI) were used to determine treatment effects for continuous and dichotomous variables, respectively.

**Results:**

Five qualified studies including 307 thoracic surgical patients were included in the current study. Among them, 65 patients were randomly allocated to the group receiving TXA administration (the TXA group); the other 142 patients were assigned to the group not receiving TXA administration (the control group). TXA significantly reduced the quantity of hemorrhage in the postoperative period (postoperative 12h: WMD = -81.90 ml; 95% CI: -139.55 to -24.26;* P* = 0.005; postoperative 24h: WMD = -97.44 ml; 95% CI: -121.44 to -73.44; *P*< 0.00001); The intraoperative blood transfusion volume (WMD = -0.54 units; 95% CI: -1.06 to -0.03; *P* = 0.04); LOS in hospital (WMD = -0.6 days; 95% CI: -1.04 to -0.16; *P* = 0.008); And there was no postoperative thromboembolic event reported in the included studies.

**Conclusions:**

The present study demonstrated that TXA significantly decreased blood loss within 12 and 24 hours postoperatively. A qualitative review did not identify elevated risks of safety outcomes such as thromboembolic events. It also suggested that TXA administration was associated with shorter LOS in hospital as compared to control. To validate this further, additional well-planned and adequately powered randomized studies are necessary.

**Supplementary Information:**

The online version contains supplementary material available at 10.1186/s13019-024-02716-9.

## Introduction

Massive bleeding remains a serious and common complication in patients undergoing thoracic surgery. Perioperative bleeding increases not only the risk of allogenic blood transfusion but also the mortality and morbidity incidences. It has been reported that the overall mortality rate in thoracic surgical patients was 17.3%, while bleeding-related mortality reached up to 15.7% [1]. Additionally, perioperative bleeding and blood transfusions increase resource utilization and medical costs [2, 3].

Surgical injury caused the release of plasminogen activator from the cytoplasmic membrane, which resulted in an increase in the local fibrinolytic processes and thus excessive bleeding [4]. Antifibrinolytic agents are commonly used during surgery to minimize bleeding and reduce exposure to blood products. Tranexamic acid (TXA) has become the most commonly used antifibrinolytic agent in clinical settings. TXA inhibits fibrinolytic activity by interacting with the lysine-binding site of plasminogen and preventing its conversion to plasmin [5]. TXA was first approved to treat menorrhagia and oral bleeding in hemophilia patients [6]. In addition, TXA has been widely administered by patients with bleeding disorders, traumatic injuries, and undergoing various types of surgery, such as cardiovascular surgery, hepatic surgery, orthopedic surgery, and obstetrics surgery [7–10]. However, accumulated evidence has suggested that TXA administration might be associated with an increased risk of thromboembolic complications [11].

The current systemic review and meta-analysis was conducted to systematically evaluate the efficacy and safety of TXA in patients undergoing thoracic surgery.

## Methods

### Search strategy

We conducted a systemic review according to the Preferred Reporting Items for Systemic Reviews and Meta-Analysis Quality of Reporting of Meta-Analysis (PRISMA) Guidelines [12]. The protocol of the present meta-analysis had been submitted to the International Prospective Systematic Reviews Registry (PROSPERO: CRD42022383262). A comprehensive and systematic search of PUBMED, Web of Science(WOS), Cochrane Library (trials), EMBASE, OVID, China National Knowledge Infrastructure (CNKI), Wanfang Data, and VIP Data from database inception to March 2023, relevant MeSH terms and keywords pertaining to tranexamic acid and thoracic surgery were combined as shown below: ((“tranexamic acid”) OR (TXA)) AND ("Thoracotomy" OR “Thoracoscope” OR “Pleuroscope” OR “VATS” OR “Video-Assisted Thoracic Surgery” OR “Esophagectomy” OR “Segmentectomy” OR “Lobectomy” OR “Thymectomy” OR “Lung” OR “Pulmonary”) AND (“randomized controlled trial” OR “controlled clinical trial” OR randomized OR randomised OR placebo OR randomly OR trial). There was no language limitation. The search strategies for all databases were described in the Supporting Information (Appendix).

### Inclusion and exclusion criteria

We included all randomized controlled trials (RCTs) in which thoracic surgery patients were randomly allocated to receive either TXA or a placebo. Among the outcomes of interest were the following: perioperative bleeding volume, intraoperative blood transfusions, the length of stay (LOS) in hospital, and the incidence of thromboembolic events. Studies released as review articles, meta-analyses, case reports, or abstracts, animal or cell studies, cardiac surgery studies, duplicate publications, or studies missing details about outcomes of interest were all excluded from consideration. Two authors (GB and LY) separately assessed the suitability of the titles as well as the abstracts of all identified papers, excluding those that were clearly ineligible. By reading the complete text, it was possible to ascertain whether the remaining works qualified for the final addition.

### Study quality assessment

The Cochrane Handbook for Systematic Reviews of Interventions and modified Jadad score were used independently by two authors (GB and LY) to evaluate the risk of bias and methodologic quality of each included trial [13, 14].

### Data abstraction

Two authors (GB and LY) extracted information from the included papers separately and created a data gathering form: (1) author, publication year, and country of included studies; (2) total number of patients, number of patients in TXA and control groups; (3) surgical procedure; (4) both groups contain results of interest. Disagreements during the data abstraction process were settled through conversation among all authors, who eventually reached a consensus.

### Statistical analysis

RevMan 5.4 (Cochrane Collaboration, Oxford, UK) was used to evaluate the pooled statistics. Dichotomous data effects were summarized as odds ratios (OR) with corresponding 95% confidence intervals (CI). While the results for continuous data were shown as the weighted mean difference (WMD) and 95% CI. Each outcome was examined for heterogeneity, and randomized-effects or fixed-effects model was used in the presence or absence of significant heterogeneity (Q-statistical test *P* <0.05). Sensitivity analyses were done by examining the influence of statistical model on estimated treatment effects, and analyses that adopted the fixed-effects model were repeated again using the randomized-effects model and vice versa. Publication bias was not assessable in the current study, as tests for funnel plot asymmetry was usually only performed when at least 10 studies were included in the meta-analysis. There were only 5 articles in the current study; therefore, tests for asymmetry was not performed. All *P* values were two-sided, and statistical significance was defined as *P* <0.05.

## Results

### Search results

**Fig 1:**
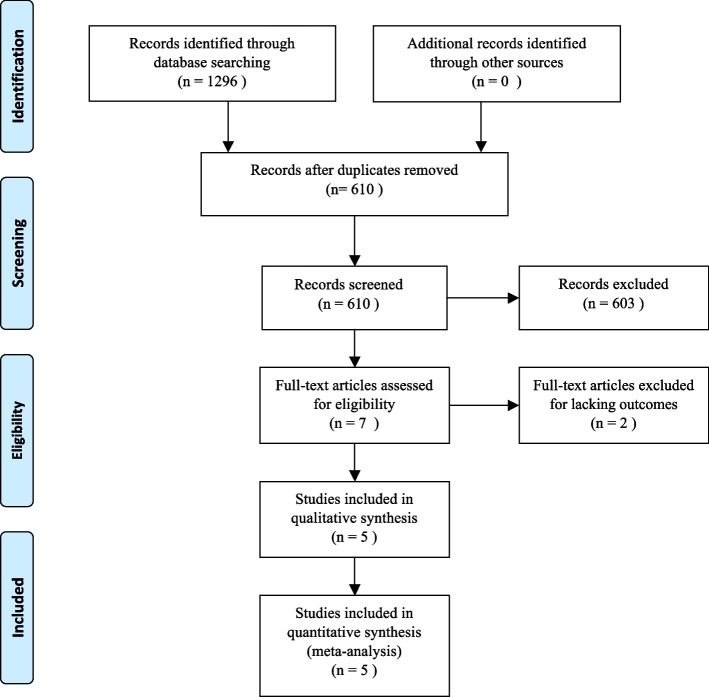
PRISMA flow diagram

Consistent with the flowchart (Fig. 1), the literature search found 1296 papers that were required to be reviewed. Finally, this meta-analysis included 307 individuals from the 5 trials [15–19] that satisfied the inclusion criteria and were examined. Descriptive analyses of these articles were presented in Table 1. Of the five literatures, three were written in English (1 from Italy, 1 from Egypt, and 1 from Israel) [15–17], and the other two were in Chinese [18, 19].

**Table 1:** Characteristic of included studies

**Study**	**Year**	**Country**	**Design**	**Surgery performed**	**Study size**	**Groups**		**Outcomes**
						**TXA**	**Control**	
Dell’Amore A et al	2012	Italy	RCT	pulmonary resection	87	5 g TXA; Thoracic topical administration *N*=44	Saline *N*=43	①②③④⑤⑦
Sabry MM et al	2018	Egypt	RCT	decortication surgery for chronic thoracic empyema, encysted effusion, or clotted hemothorax	70	3 g TXA; Thoracic topical administration *N*=35	Saline *N*=35	①②③⑤⑦
Kuint R et al	2020	Israel	RCT	bronchoscopy and TBLB	50	0.5gTXA; instilled in the target lobar bronchus *N*=26	Saline *N*=24,	⑥⑦
Wang LT et al	2021	China	RCT	lobectomy	60	2gTXA; Thoracic topical administration *N*=20	Saline *N*=20	①②④⑤⑦
						3gTXA; Thoracic topical administration *N=20*		
Hu Y et al	2006	China	RCT	lobectomy and esophagectomy	40	10mg/ kg as a bolus; 1mg·kg^-1^·h^-1 ^continuous infusion during the surgery * N=20*	Saline *N*=20	②⑥

*RCT* randomized controlled trial, *TXA* tranexamic acid, *TBLB* transbronchial lung biopsies

①= The blood loss on post-operative 12h

②= The blood loss on post-operative 24h

③= Blood transfusion

④= Post-operative thromboembolic events

⑤= Hospital stay

⑥= Operative blood loss

⑦= Thromboembolic events

### Included trials characteristics

According to the results shown in Table 1, among the five trials, one included patients receiving pulmonary resection [15], one included patients undergoing lung decortication surgery [16], one included patients undergoing bronchoscopy and transbronchial lung biopsies (TBLB) [17], one included only patients undergoing lobectomy [18], and one included patients undergoing lobectomy and esophagectomy [19]. The 5 eligible trials involved 307 patients, of whom 165 were allocated to the TXA group and 142 to the control group (placebo). In separate studies, TXA was delivered at varying dosages. The research conducted by Wang et al. examined two dosages of TXA administration [18]; hence, it was divided into two distinct groups.

### Risk of bias in included studies

**Fig 2:**
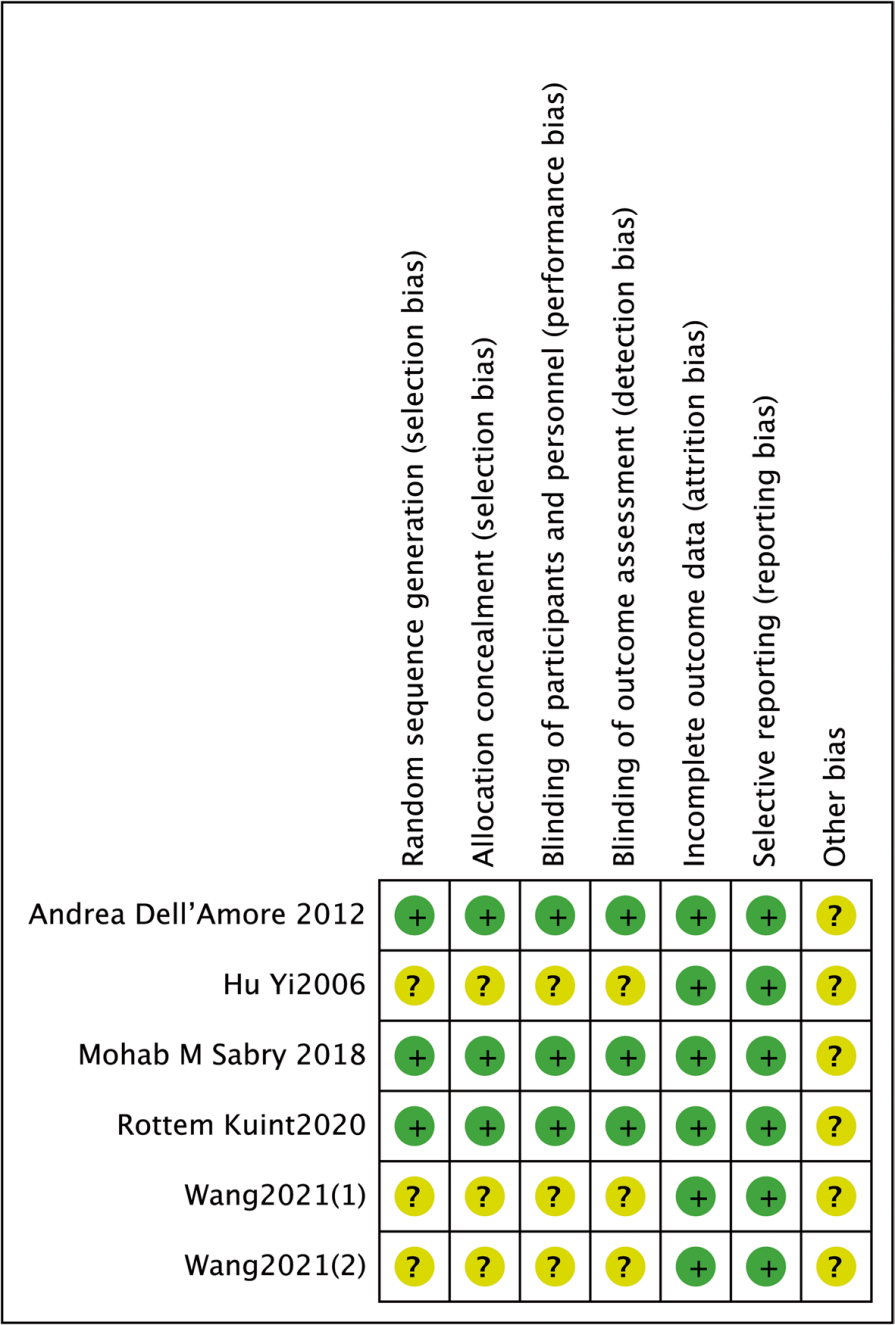
Risk of bias of studies

**Fig 3:**
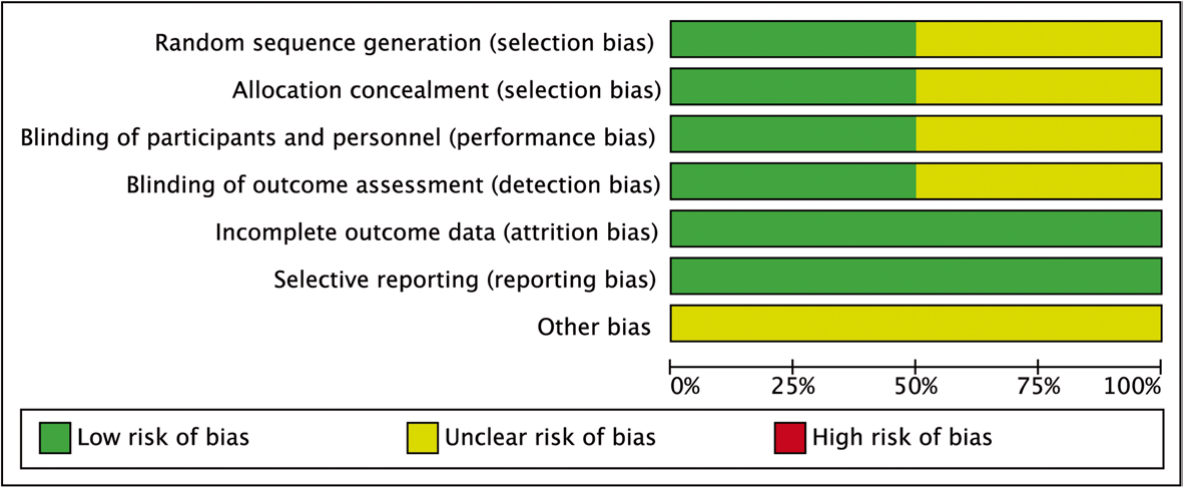
Risk of bias graph

The quality analysis of the included RCTs was illustrated in Fig. 2. There was some concern about selection bias due to the fact that some trials did not clearly describe the details of the random generation process and allocation concealment. In addition, a graphical overview of the evaluations made on each item of methodological quality for each of the included trials was shown in Fig. 3. As indicated in Table 2, three of the five included trials had Jadad scores greater than 3 and were deemed high-quality RCTs [15–17].

**Table 2:** Quality assessment of included studies

Study	Sample size	Jadad score			
		Randomization	Blindness	Withdrawals	Total
Dell’Amore 2012	87	2	2	1	5
Sabry 2018	70	2	2	0	4
Kuint 2020	50	2	2	1	5
Wang 2021	60	1	1	0	2
Hu 2006	40	1	1	0	2

### Perioperative bleeding volume

**Fig 4:**
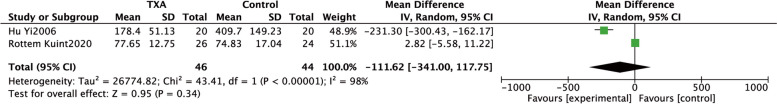
Intraoperative bleeding volume

We investigated the effect of TXA on intraoperative and postoperative hemorrhage. As shown in Table 1, 2 trials (2 comparisons, 90 patients) reported intraoperative blood loss in patients undergoing bronchoscopy [17, 19], TBLB, lobectomy, and esophagectomy surgery, respectively. Meta-analysis demonstrated that the amount of intraoperative blood loss did not differ between TXA and control groups [(WMD = -111.62 ml; 95% CI -341.00 to -117.75; *P* = 0.34) with heterogeneity (I^2^ = 98%, *P* <0.00001)]. As shown in Fig. 4.

**Fig 5:**
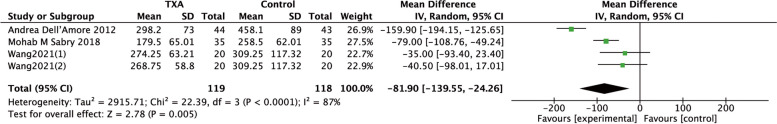
Postoperative 12h bleeding volume

Three trials (4 comparisons, 217 patients) reported postoperative 12-hour bleeding volumes in patients undergoing pulmonary resection, lobectomy, and decortication surgery [15, 16, 18], respectively. According to the meta-analysis, TXA dramatically reduced the amount of postoperative hemorrhage for 12 hours [(WMD = -81.90 ml; 95% CI: -139.55 to -24.26; *P =* 0.005) with heterogeneity (I^2^ = 87%, *P* < 0.0001)]. As shown in Fig. 5.

**Fig 6:**
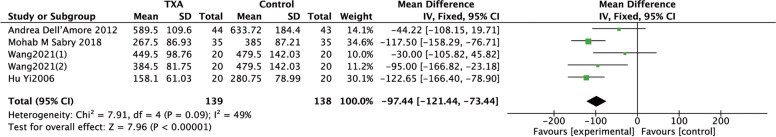
Postoperative 24h bleeding volume

Four trials (5 comparisons, 257 patients) reported postoperative 24-hour bleeding volumes in patients undergoing pulmonary resection, lobectomy, esophagectomy, and decortication surgery, respectively [15, 16, 18, 19]. According to the meta-analysis, TXA dramatically dropped the amount of postoperative hemorrhage for 24 hours [(WMD = -97.44 ml; 95% CI: -121.44 to -73.44; *P* < 0.00001) with heterogeneity (I^2^ = 49%, *P* = 0.09)]. As shown in Fig. 6.

### Intraoperative blood transfusion

**Fig 7:**
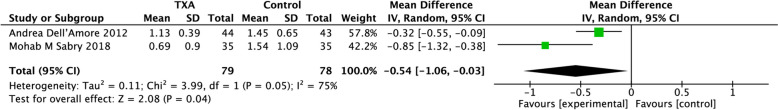
Intraoperative blood transfusion

As illustrated in Table 1, in 2 trials (2 comparisons, 157 patients), the intraoperative blood transfusion volume was reported in patients undergoing pulmonary resection and decortication surgery, respectively [15, 16]. Meta-analysis revealed that TXA substantially decreased the volume of intraoperative blood transfusion [(WMD = -0.54 units; 95% CI: -1.06 to -0.03; *P* = 0.04) with heterogeneity (I^2^ = 75%, *P* = 0.05)]. As shown in Fig. 7.

### LOS in hospital

**Fig 8:**
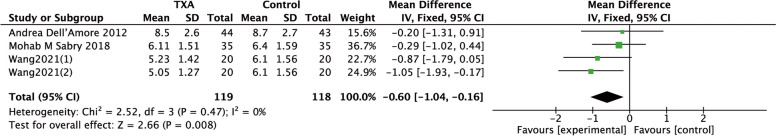
Length of stay in hospital

Three studies reported the LOS in hospital [15, 16, 18]. Meta-analysis demonstrated that the TXA group significantly reduced the LOS in hospital compared to the control group [(WMD = -0.6 days; 95% CI: -1.04 to -0.16; *P* = 0.008) with heterogeneity (I^2^ = 0%, *P* = 0.47)]. As shown in Fig. 8.

### The incidence of thromboembolic events

The outcomes of thromboembolic events were reported in four studies [15–18], and none of them occurred postoperatively.

## Discussion

To our knowledge, this is the first study dedicated to systematically evaluate the efficacy and safety of TXA in patients undergoing thoracic surgery.

It is well known that the lungs contribute to platelet biogenesis [20]. As a result of the platelet shortage brought on by lung resection, blood clotting becomes defective, which is the most frequent reason for hemorrhage after cardiothoracic surgical procedures [21]. TXA, a lysine analogue, became the most frequently prescribed antifibrinolytic drug following the withdrawal of aprotinin from the market [22]. It binds to plasminogen and blocks the release of active plasminogen mediated by tissue-type plasminogen activator (t-PA). By blocking the binding of fibrinogen to fibrin at the lysine binding site, fibrin polymers have greater resistance to fibrinolysis [23]. TXA is administered in a variety of ways, including oral, topical, and intravenous [24]. Through the aforementioned methods of administration, TXA has been shown to be effective in reducing bleeding and the need for blood transfusions in patients undergoing surgical procedures [25–27].

This is a comprehensive evaluation of five studies of TXA topical administration. Katharine Ker et al. suggested that topical application of TXA could reduce surgical blood loss by about one-third in a series of surgical procedures [28]. However, the effects may vary with different types of surgery. We can conclude from these studies that TXA does not significantly reduce intraoperative blood loss, but it does significantly reduce postoperative (postoperative 12h and postoperative 24h) bleeding as measured by total thoracic drainage. However, there is a practical situation that needs to be considered. The blood loss during thoracoscopic major pulmonary resections ranges from 10 to 400 mL [29], while the blood loss during thoracotomy is even greater. Whether the difference in blood loss between the two groups has clinical significance is still debatable and needs to be verified by more clinical trials.

At 12 hours after surgery, Dell' Amore et al. and Sabry et al. reported that the blood loss was significantly decreased through thoracic drainage in the TXA group [15, 16], while Wang et al.'s results were less significant [18]. It could be argued that the high heterogeneity calculated for hemoglobin drop (I^2^ = 87%) could be related to unreliable results. The direction and magnitude of the effects, the *P* value (*P* < 0.00001), and the 95% CI dictate that the presented results are reliable. Consequently, this meta-analysis showed that the postoperative blood loss in patients undergoing thoracic surgery with the administration of TXA was significantly lower than in the patients who did not receive the drug. TXA had a statistically significant lesser amount of postoperative blood loss up to 24 hours. These results were consistent with Mieke et al. [30], whose meta-analysis indicated the perioperative estimated blood loss was lower in patients receiving a single dose of intravenous TXA.

Tranexamic acid application is gaining widespread acceptance in the field of cardiac surgery. Habbab et al. suggested that intrapericardial use of TXA decreased postoperative bleeding without raising the danger of postoperative complications [31]. Additionally, thoracic operations should receive similar consideration. TXA is rarely administered intravenously in thoracic surgery and is predominantly administered locally, which may be related to the pleura, a natural barrier that minimizes the absorption of TXA into systemic circulation and avoids harmful side effects. TXA is an inexpensive and effective way to reduce the incidence of postoperative bleeding compared to the frequently used, expensive local thrombosis inhibitors.

Additional factors associated with the treatment of the patients, such as the LOS in hospital was also evaluated in this meta-analysis. According to Henry A. Pitt et al. [32], reducing LOS in hospital is an effective cost-cutting strategy that may improve patient satisfaction. Although an assessment of cost-effectiveness was outside the scope of this study and TXA shortened the LOS, it could ultimately reduce overall healthcare costs in combination with the reduction in blood transfusion volume.

Although many studies had shown that intravenous administration of TXA reduced postoperative bleeding, Gomez-Barrena et al. reported that topical administration of TXA showed non-inferiority and no safety concerns compared to intravenous administration [33]. The major conclusions of this meta-analysis are consistent with those of previous research and comprehensive reviews. The TXA group experienced less blood transfer, less blood loss, and a shorter LOS in hospital than the control group.

This meta-analysis had certain limitations. Firstly, the amount of TXA used in various studies varies (Table 1). The most appropriate dosage for intravenous or topical delivery was not presently agreed upon. Therefore, more research was required to determine the ideal TXA dosage. Secondly, given few studies by intravenous administration, we were unable to perform subgroup analyses that may explain differences in responses to TXA. Larger trials are needed to validate these findings. Thirdly, there was no standard technique for evaluating postoperative bleeding, which was measured by chest drainage, and the computation of chest drainage differed between studies. For example, Sabry et al. calculated the amount of bleeding by visually differentiating between serosanguinous pleural fluid drainage and blood [16], whereas Dell Amore et al. did not describe in detail the calculation of chest tube drainage to estimate the amount of blood loss [15]. Lastly, despite the fact that our literature search was comprehensive, including eight databases, there were only five trials ultimately included for analysis. As a result, this research may be affected by the "small study effect," whereby small trials have a tendency to exaggerate therapy effects because of methodological differences. Larger, well-designed clinical trials are still required to evaluate the use of TXA in patients undergoing thoracic surgery.

## Conclusions

The present study demonstrated that TXA significantly decreased blood loss within 12 and 24 hours postoperatively. A qualitative review did not identify elevated risks of safety outcomes such as thromboembolic events. It also suggested that TXA administration was associated with shorter LOS in hospital as compared to control. To validate this further, additional well-planned and adequately powered randomized studies are necessary.

### Supplementary Information

**Additional file1.** Search strategy

## Data Availability

No datasets were generated or analysed during the current study.
