# Supplementary material for: Efficacy and safety of tranexamic acid in patients undergoing thoracic surgery: a systematic review and PRISMA-compliant meta-analysis
Source: J Cardiothorac Surg. 2024 Apr 9;19:195. doi: 10.1186/s13019-024-02716-9 (PMC11005289; doi:10.1186/s13019-024-02716-9)
Supplement: Supplementary file 1 — Additional file1. Search strategy [file 13019_2024_2716_MOESM1_ESM.docx]

| **Search strategies** | **Results** |
| --- | --- |
| **Pubmed**  #1: "Thoracotomy"[MeSH Terms] OR "Thoracotomies"[Title/Abstract] OR "Thoracoscopes"[MeSH Terms] OR "Thoracoscope"[Title/Abstract] OR "Pleuroscopes"[Title/Abstract] OR "Pleuroscope"[Title/Abstract] OR "thoracic surgery, video assisted"[MeSH Terms] OR "surgeries video assisted thoracic"[Title/Abstract] OR "surgery video assisted thoracic"[Title/Abstract] OR "thoracic surgery video assisted"[Title/Abstract] OR "Video-Assisted Thoracic Surgeries"[Title/Abstract] OR "video assisted thoracoscopic surgery"[Title/Abstract] OR "surgery video assisted thoracoscopic"[Title/Abstract] OR "thoracoscopic surgeries video assisted"[Title/Abstract] OR "thoracoscopic surgery video assisted"[Title/Abstract] OR "video assisted thoracoscopic surgery"[Title/Abstract] OR "Video-Assisted Thoracoscopic Surgeries"[Title/Abstract] OR "video assisted thoracic surgery"[Title/Abstract] OR "video assisted thoracic surgery"[Title/Abstract] OR "VATS"[Title/Abstract] OR "VATSs"[Title/Abstract] OR "Segmentectomy"[Title/Abstract] OR "Lobectomy"[Title/Abstract] OR "Esophagectomy"[Title/Abstract] OR "thymectomy"[Title/Abstract] OR "Pulmonary"[Title/Abstract] OR "lung"[Title/Abstract] OR "VATS"[Title/Abstract]  #2: "tranexamic acid"[Title/Abstract] OR "TXA"[Title/Abstract]  #3: "randomized controlled trial"[Title/Abstract] OR "controlled clinical trial"[Title/Abstract] OR "randomized"[Title/Abstract] OR "placebo"[Title/Abstract] OR "randomly"[Title/Abstract] OR "trial"[Title/Abstract] OR "randomised"[Title/Abstract]  #4: #1 AND #2 AND #3 | 203 |
| **Embase**  #1: 'thoracotomy'/exp OR 'thoracoscope'/exp OR 'video assisted thoracoscopic surgery'/exp  #2: thoracotomies:ab,ti OR thoracoscope:ab,ti OR pleuroscopes:ab,ti OR pleuroscope:ab,ti OR 'video assisted thoracoscopic surgery':ab,ti OR segmentectomy:ab,ti OR lobectomy:ab,ti OR esophagectomy:ab,ti OR thymectomy:ab,ti OR pulmonary:ab,ti OR lung:ab,ti  #3: 'surgeries video assisted thoracic':ab,ti OR 'surgery video assisted thoracic':ab,ti OR 'thoracic surgery video assisted':ab,ti OR 'video-assisted thoracic surgeries':ab,ti OR 'surgery video assisted thoracoscopic':ab,ti OR 'thoracoscopic surgeries video assisted':ab,ti OR 'thoracoscopic surgery video assisted':ab,ti OR 'video assisted thoracoscopic surgery':ab,ti OR 'video-assisted thoracoscopic surgeries':ab,ti OR 'video assisted thoracic surgery':ab,ti OR 'vatss':ab,ti  #4: 'tranexamic acid':ab,ti OR txa:ab,ti  #5: 'randomized controlled trial':ab,ti OR 'controlled clinical trial':ab,ti OR randomized:ab,ti OR randomised:ab,ti OR placebo:ab,ti OR randomly:ab,ti OR trial:ab,ti  #6: #1 OR #2 OR #3  #7: #4 AND #5 AND #6 | 279 |
| **Cochrane library(Trials)**  #1 MeSH descriptor: [Thoracotomy] explode all trees 654  #2 MeSH descriptor: [Thoracoscopes] explode all trees 4  #3 MeSH descriptor: [Thoracic Surgery, Video-Assisted] explode all trees 380  #4 thoracotomies 113  #5 thoracoscope 92  #6 pleuroscopes 1  #7 pleuroscope 3  #8 "video assisted thoracoscopic surgery" 847  #9 "surgery video assisted thoracic" 25  #10 "thoracic surgery video assisted" 407  #11 "video-assisted thoracic surgeries" 9  #12 "surgery video assisted thoracoscopic" 80  #13 "thoracoscopic surgeries video assisted" 1  #14 "thoracoscopic surgery video assisted" 4  #15 "video assisted thoracoscopic surgery" 847  #16 "'video-assisted thoracoscopic surgeries" 9  #17 "video assisted thoracic surgery" 364  #18 vatss 1  #19 segmentectomy 352  #20 lobectomy 1863  #21 esophagectomy 1412  #22 thymectomy 165  #23 pulmonary 64911  #24 "lung" 86608  #25 "surgeries video assisted thoracic" 1  #26 #1 OR #2 OR #3 OR #4 OR #5 OR #6 OR #7 OR #8 OR #9 OR #10 OR #11 OR #12 OR #13 OR #14 OR #15 OR #16 OR #17 OR #18 OR #19 OR #20 OR #21 OR #22 OR #23 OR #24 OR #25 123164  #27 "tranexamic acid" 3718  #28 "TXA" 1223  #29 #27 OR #28 3776  #30 "randomized controlled trial" 622268  #31 "controlled clinical trial" 180138  #32 "randomized" 1145894  #33 "randomised" 1145894  #34 "placebo" 368157  #35 "randomly" 303519  #36 "trial" 1310715  #37 #30 OR #31 OR #32 OR #33 OR #34 OR #35 OR #36 1591726  #38 #26 AND #29 AND #37 355 | 304 |
| **Web of science**  #1: **Thoracotomy (主题) or Thoracoscopes (主题) or "Thoracic Surgery, Video-Assisted" (主题) or thoracoscope (主题) or pleuroscopes (主题) or pleuroscope (主题) or "video assisted thoracoscopic surgery" (主题) or "surgery video assisted thoracic" (主题) or "thoracic surgery video assisted" (主题) or "video-assisted thoracic surgeries" (主题) or "surgery video assisted thoracoscopic" (主题) or "thoracoscopic surgeries video assisted" (主题) or "thoracoscopic surgery video assisted" (主题) or "video assisted thoracoscopic surgery" (主题) or "video-assisted thoracoscopic surgeries" (主题) or "video assisted thoracic surgery" (主题) or vatss (主题) or segmentectomy (主题) or lobectomy (主题) or esophagectomy (主题) or thymectomy (主题) or pulmonary (主题) or lung (主题) or "surgeries video assisted thoracic" (主题) or thoracotomies (主题)**  **#2: "****tranexamic acid" (主题) or TXA (主题)**  **#3:** **"randomized controlled trial" (主题) or "controlled clinical tria" (主题) or randomized (主题) or randomised (主题) or placebo (主题) or randomly (主题) or****trial (主题)**  **#4: #1 AND #2 AND #3** | 265 |
| **OVID**  #1: Thoracotomy.mp. or exp Thoracotomy/  #2: Thoracoscopes.mp. or exp Thoracoscopes/  #3: "Thoracic Surgery, Video-Assisted".mp. or exp Thoracic Surgery, Video-Assisted/  #4: (segmentectomy or lobectomy or esophagectomy or thymectomy or pulmonary or lung).ab.  #5: ("surgeries video assisted thoracic" or thoracotomies or thoracoscope or "surgery video assisted thoracic" or "thoracic surgery video assisted" or "video-assisted thoracic surgeries" or "surgery video assisted thoracoscopic" or "thoracoscopic surgeries video assisted" or "thoracoscopic surgery video assisted" or "video assisted thoracoscopic surgery" or "video-assisted thoracoscopic surgeries" or "video assisted thoracic surgery" or vatss).ab.  #6: #1 OR #2 OR #3 OR #4 OR #5  #7: ("tranexamic acid" or TXA).ab.  #8: ("randomized controlled trial" or randomised or "controlled clinical trial" or randomized or placebo or randomly or trial).ab  #9: #6 AND #7 AND #8 | 197 |
| **CNKI**  (SU %= '胸科手术' OR SU %= '开胸手术' OR SU %= '胸腔镜' OR SU %= '肺癌根治术' OR SU %= '肺叶切除' OR SU %= '肺段切除' OR SU %= '肺楔形切除' OR SU %= '胸腺切除' OR SU %= '食管切除' OR SU %= '食管癌' ) AND SU %= '氨甲环酸' | 4 |
| **WANFANG**  (主题:(胸科手术) or 主题:(开胸手术) OR 主题:(胸腔镜手术) or 主题:(胸腔镜肺癌根治术) or 主题:(开胸肺癌根治术) or 主题:(肺叶切除术) or 主题:(肺段切除术) or 主题:(胸腺切除) or 主题:(食管切除) or 主题:(食管癌)) and 主题:(氨甲环酸) | 42 |
| **VIP**  (M= 胸科手术 OR M= 开胸手术 OR M= 胸腔镜 OR M= 肺癌根治术 OR M= 肺叶切除 OR M= 肺段切除 OR M= 肺楔形切除 OR M= 胸腺切除 OR M= 食管切除 OR M= 食管癌 ) AND M= 氨甲环酸 | 2 |
